# Sleep deprivation in adolescent mice impairs long-term memory till early adulthood via suppression of hippocampal astrocytes

**DOI:** 10.1093/sleep/zsae143

**Published:** 2024-06-27

**Authors:** Ji-Yun Kang, Jin-Seok Lee, Jing-Hua Wang, Chang-Gue Son

**Affiliations:** Institute of Bioscience & Integrative Medicine, Daejeon Hospital of Daejeon University, Daejeon, South Korea; Institute of Bioscience & Integrative Medicine, Daejeon Hospital of Daejeon University, Daejeon, South Korea; Research Center for CFS/ME, Daejeon Hospital of Daejeon University, Daejeon, Republic of Korea; Institute of Bioscience & Integrative Medicine, Daejeon Hospital of Daejeon University, Daejeon, South Korea; Research Center for CFS/ME, Daejeon Hospital of Daejeon University, Daejeon, Republic of Korea; Institute of Bioscience & Integrative Medicine, Daejeon Hospital of Daejeon University, Daejeon, South Korea; Research Center for CFS/ME, Daejeon Hospital of Daejeon University, Daejeon, Republic of Korea

**Keywords:** sleep deprivation, adolescence, early adulthood, hippocampus, neurogenesis, astrocyte

## Abstract

Sleep deficiency is a rampant issue in modern society, serving as a pathogenic element contributing to learning and memory impairment, with heightened sensitivity observed in children. Clinical observations suggest that learning disabilities associated with insufficient sleep during adolescence can persist through adulthood, but experimental evidence for this is lacking. In this study, we examined the impact of early-life sleep deprivation (SD) on both short-term and long-term memory, tracking the effects sequentially into adulthood. We employed a modified multiple-platform method mouse model to investigate these outcomes. SD induced over a 14-day period, beginning on postnatal day 28 (PND28) in mice, led to significant impairment in long-term memory (while short-term memory remained unaffected) at PND42. Notably, this dysfunction persisted into adulthood at PND85. The specific impairment observed in long-term memory was elucidated through histopathological alterations in hippocampal neurogenesis, as evidenced by bromodeoxyuridine (BrdU) signals, observed both at PND42 and PND85. Furthermore, the hippocampal region exhibited significantly diminished protein expressions of astrocytes, characterized by lowered levels of aquaporin 4 (AQP4), a representative molecule involved in brain clearance processes, and reduced protein expressions of brain-derived neurotrophic factors. In conclusion, we have presented experimental evidence indicating that sleep deficiency-related impairment of long-term memory in adolescence can endure into adulthood. The corresponding mechanisms may indicate that the modification of astrocyte-related molecules has led to changes in hippocampal neurogenesis.

## Graphical Abstract

**Graphical Abstract:**
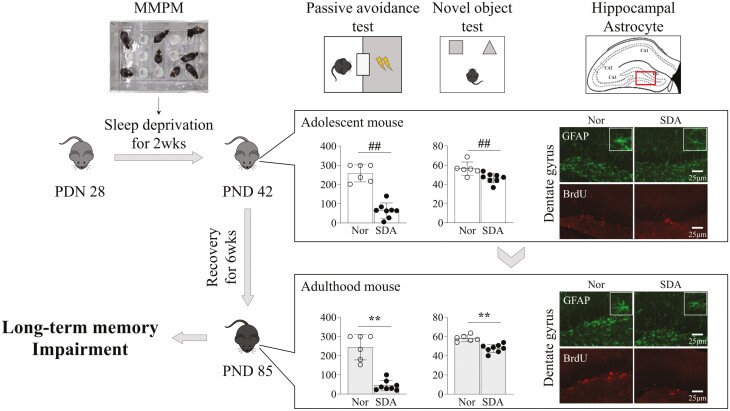

**Statement of Significance**

Researchers utilized mice, with their shorter lifespan compared to humans, to explore the overall impact of sleep on life. sleep deprivation (SD) was induced in 4-week-old mice (equivalent to human teenagers) for 14 days, and the effects were evaluated when they reached 12 weeks old, akin to humans in their early adulthood. The study revealed that SD during adolescent mice led to long-term memory impairment that persisted into adulthood. This impairment in long-term memory could be linked to reduced neurogenesis caused by decreased hippocampal astrocyte-related molecules. This study introduces a novel mechanism explaining brain dysfunction and behavioral disorders resulting from SD in adolescent mice.

In light of changes in social environments and lifestyles, sleep-related issues have emerged as a significant global health concern [1]. Research indicates that approximately 62% of adults worldwide do not meet the recommended sleep duration of 8 hours [2]. Insufficient sleep has been linked to impaired brain functions, specifically affecting emotional expression, reward processing, and memory formation [3, 4]. Clinical studies have identified sleep disruption and chronic short sleep as potential risk factors for neurodegenerative disorders, including Alzheimer’s disease (AD) and Parkinson’s disease [5, 6].

Particularly noteworthy is the profound impact of both sleep quantity and quality on memory formation in the young population [7]. Adolescents, in particular, are highly susceptible to disrupted sleep patterns due to its crucial role in both physical and mental development [8, 9]. Under identical sleep deficiency conditions, teenagers (aged 13 to 16 years old) exhibited notably higher anxiety scores (1.47-fold) and experienced a decline in learning competency compared to early adults [10, 11]. Moreover, teenagers (aged 12 to 18 years old) experiencing insomnia demonstrated a substantially elevated incidence of depression (2.2-fold) and substance abuse (1.25-fold) upon reaching young adulthood (aged 18 to 25 years old) [12]. Research findings indicate that reduced sleep duration among middle and high school students correlated with decreased academic performance, specifically in areas related to learning and memory [13]. Additionally, individuals experiencing insufficient sleep during early adulthood had a 30% higher likelihood of developing dementia compared to those with normal sleep patterns (defined as 7 hours) [14].

During the sleep cycle, the process of consolidation in the hippocampus facilitates the transformation of learned information into long-term memory [15]. Multiple clinical studies have found that sleep deprivation (SD) in children led to long-term memory deficits as well as decreases in hippocampal gray matter volume and neuroplasticity [16, 17]. Hippocampal neurogenesis plays a crucial role in learning and memory across various mammalian species, including humans [18]. More recently, it has been recognized that hippocampal neurogenesis depends significantly on the integrity of brain glial cells, particularly astrocytes [19].

Previous studies suggest that inadequate sleep in teenagers can lead to persistent memory impairment in adulthood [20, 21]. However, there is a lack of experimental data to substantiate these claims. In this study, our objective was to investigate the extent to which SD in adolescent mice (SDA) impacts memory-related brain function in adulthood and to explore the underlying mechanisms.

## Methods

### Animal care and experimental designs

A total of 48 specific pathogen-free male C57BL/6J mice, aged 3 weeks and weighing 9 to 10 g, were procured from Dae Han Bio Link (Co., Ltd., Eumseong, Korea). The mice had ad libitum access to water and food pellets (Cargill Agri Purina, Seongnam, Korea) and were housed in a room maintained at 23 ± 1°C on a 12 h:12 h light–dark cycle (lights on at 09:00 a.m. [Zeitgeber Time 0]). The animal care and experimental protocols were approved by the Institutional Animal Care and Use Committee of Daejeon University (DJUARB2021-014) and conducted following the Guide for the Care and Use of Laboratory Animals published by the USA National Institutes of Health (NIH). After adaptation for 7 days, they were used in the experiment.

**Figure 1.**
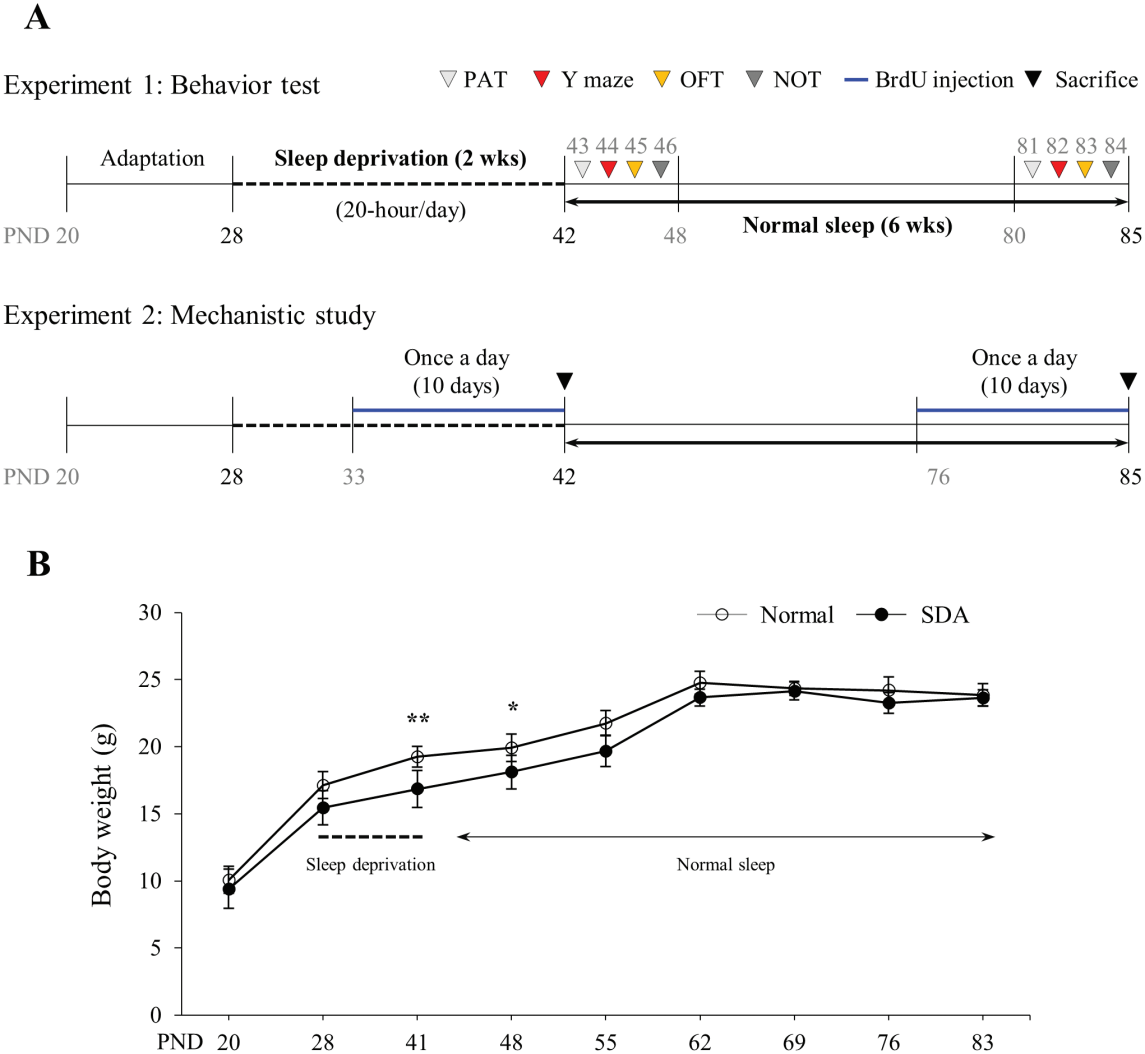
Experimental design of this study. In experimental study 1 (A), behavioral tests were conducted on postnatal day 42 (PND42) following a 14-day period of sleep deprivation. Subsequently, additional behavioral tests were administered on PND85 after a 6-week period of normal sleep. In experimental study 2 (B), bromodeoxyuridine (BrdU) was injected daily for 10 days before the animals were euthanized. Blood and brain tissue samples were collected at both PND42 and PND85 to investigate the underlying mechanisms. Bodyweight measurements were recorded from postnatal day 20 to PND85 (C). The data are expressed as the means ± standard deviations (*n* = 8). *p < .05 and **p < .01 compared with the normal group

In experiment 1, a behavioral test was conducted. The mice were randomly divided into two groups: normal and SD (each consisting of six and eight mice, respectively). Two mice were excluded from the analysis because of a wound observed on the dorsal region of the mice during the adaptation period. Starting from postnatal day 28 the mice in the sleep-deprivation group underwent 20 hours of SD per day, beginning at 6:00 pm, for a total of 14 days. During the recovery condition, the mice were permitted to sleep freely in their existing home cages for 4 hours. Meanwhile, the normal mice were placed in equivalent home cages. Following the completion of the SD protocol, behavioral tests were carried out on postnatal day 42 (PND42), as outlined in the subsequent sections. After 6 weeks of adequate sleep, the same animals underwent a repeat of the behavioral tests at PND85 (Figure 1A).

In experiment 2, mechanistic studies were conducted following the same SD protocol as in experiment 1. The mice were divided randomly into four groups: normal and SD groups at PND42 and PND85, each comprising eight mice. Intraperitoneal injections of bromodeoxyuridine (BrdU, 50 mg/kg) were administered daily for 10 days, starting from PND 33 to 42 and PND 76 to 85, respectively. By this time, the majority of BrdU-labeled cells represented newly generated brain cells. We sacrificed the 24-hour-after-last injection timepoint for evaluating cell proliferation in mice (*n* = 3 per group). In addition, the mice were euthanized for blood collection and brain dissection at both postnatal 42 and 85 days, respectively (*n* = 5 per group, Figure 1A). Although the sleep-deprived group exhibited decreased body weight during the SD period, they showed rapid recovery during the subsequent growth process. Notably, there was no significant difference observed in the growth period compared to the normal group (Figure 1B).

### Modified multiple platform methods for inducing SD

The modified multiple platform method (MMPM) was utilized to establish an animal SD model, as previously described [22]. In summary, to induce SD, mice were housed in cages measuring 27 × 42 × 18 cm, equipped with multiple platforms (15 cylinders, each 3.5 cm in diameter) filled with tap water (maintained at 23 ± 1°C, with water level kept 1 cm below the platform surface) and mice could move and perch on the platforms. When they enter rapid eye movement (REM) sleep and experience muscle atonia, they fall into the water and awaken [23]. It has been demonstrated that the MMPM is more effective in depriving rodents of REM sleep than non-REM (NREM) sleep [24, 25]. The water was refreshed daily during the induction period. Provision of food and water occurred through a grid placed atop the water tank.

### Passive avoidance test

Long-term memory, assessed through fear-conditioning, was evaluated using a passive avoidance test (PAT). The test apparatus consisted of a shuttle box divided into a light and a dark compartment of equal size, separated by a partition with a door. During the training session, the mouse was placed in the well-lit compartment, facing away from the entrance to the dark compartment. The door opened 10 seconds later, and upon entry into the dark compartment, an inescapable foot shock (0.5 mA, 5 seconds) was administered through the grid floor. Subsequently, the passive-avoidance response was evaluated at 24 hours and 37 days (PND42 and PND85, respectively). Each mouse was reintroduced into the brightly lit compartment, and the latency time for reentering the dark compartment was recorded. No foot shock was administered during the test. The maximum allowable latency time for reentry was set at 300 seconds [26]. The PAT was performed at 21:00–22:00 at PND 43 and PND 81, respectively.

### Novel object test

Recognition memory was assessed using a novel object test (NOT), adapted from the procedure outlined by Marianne Leger [27]. Prior to the training session, mice were acclimated to an open field (40 × 40 × 30 cm) for 10 minutes within 24 hours. During the training session, each mouse had a 10-minute exposure to identical objects (2 × 4 × 10 cm) placed in the left and right corners of the arena. Following the training, mice returned to their home cages and had a 6-hour retention interval for rest and sleep.

In the testing session, each mouse was reintroduced into the field where one familiar object was replaced with a novel object (2 × 4 × 10 cm). The testing session was recorded on video, and the mice were allowed 10 minutes for exploration. At red light for 50 lux illuminations, we meticulously recorded the time each mouse spent exploring each object in the video. Exploration was defined as the mouse’s nose being within 2 cm of and directed toward the object. Instances where the animal propped itself on the object were not considered exploration time for that object. The discrimination ratio was calculated by dividing the time spent exploring the novel object by the total exploration time of either object. The NOT was performed at 21:00–22:00 at PND 46 and PND 84, respectively.

### Y maze test

Short-term spatial memory was evaluated using a Y maze test, a method previously described [28]. The Y maze utilized in the study was constructed from acrylic material and featured three arms measuring 60 × 60 × 15 cm, each separated by 120°C. In red light for 50 lux illuminations condition, the assessment process involved a 2-minute encoding trial where one arm was blocked, followed by a 1-minute interval, and concluding with a 2-minute test session. The starting arm remained consistent for both encoding and testing phases, with the exposed arm during the encoding trial considered the familiar arm and the blocked arm designated as the novel arm. A mouse was deemed to be on an arm if its entire body crossed the entrance line.

Upon completion of the test session, the mouse was returned to its home cage. The maze was cleaned with 70% alcohol, allowed to dry, and prepared for the next mouse. The short-term spatial memory performance was quantified based on the latency time taken by the mouse to explore the novel arm in the Y maze. The Y maze was performed at 21:00–22:00 at PND 44 and PND 82, respectively.

### Open field test

Anxiety-like behavior and locomotor activity were evaluated using an open-field test as previously described [29]. The plastic box for the open field apparatus was contained on the square side (40 × 40 × 30 cm), and the center of the field was distinguishable in the recording software. To evaluate the hyperactivity conditions, each mouse was placed in the center of the field. Their total traveled distance was subsequently recorded for 8 minutes at red light for 50 lux illumination using a video camera connected to the corresponding software (Smart Junior, Bang Na, Thailand). The open field test (OFT) was performed at 21:00–22:00 at PND 45 and PND 83, respectively.

### Blood collection and brain tissue preparation

Mice were sacrificed under CO_2_ anesthesia on PND42 and PND85, respectively. Blood was collected according to the instructions of the Animal Management and Use Committee. Serum was collected after centrifugation at 3000 rpm for 15 minutes at 25°C and stored at −80°C. After transcranial perfusion, the brains of three mice in each group were fixed in a 4% paraformaldehyde (PFA) solution for immunofluorescent staining. The hippocampus was immediately isolated from the whole brain of the remaining five mice, and then the samples were stored at −80°C. Subsequently, five mouse brains were pooled in RIPA buffer to homogenize the hippocampus in preparation for biochemical analyses, including enzyme-linked immunosorbent assay (ELISA) and western blotting, and the pooling brain samples were assessed in triplicate. Protein concentrations were determined using a Bicinchoninic Acid (BCA) protein assay kit by measuring the absorbance at 560 nm using a spectrophotometer (Molecular Devices Corp., Sunnyvale, CA, USA).

### Determination of cytokines and corticosterone

ELISA was performed to evaluate the hippocampal cytokines. The levels of interleukin 6 (IL-6), IL-10, tumor necrosis factor-α (TNF-α), and TGF-β in the hippocampus tissue were detected using commercial ELISA kits according to the manufacturer’s instructions (BD Bioscience). The serum level of corticosterone was measured using commercial enzyme-linked immunosorbent assay kits according to the manufacturer’s instructions (catalog no. K014-H5). The absorbance at 450 and/or 570 nm was measured using a spectrophotometer (Molecular Devices).

### Western blot analysis

A western blot was performed to evaluate the hippocampal neurogenesis-related markers (doublecortin; DCX and neuronal nuclear protein; NeuN), glia cell expression (glial fibrillary acidic protein; GFAP and ionized calcium-binding adapter molecule 1; Iba-1), markers that may be related to astrocytes (Janus kinase 1; JAK1 and signal transducer and activator of transcription 3; STAT3), and expression of these factors (brain-derived neurotrophic factor; BDNF, cAMP response element-binding protein; CREB, aquaporin 4; AQP4, and p38 mitogen-activated protein kinase; p38) in the hippocampus. The brain tissues were denatured by boiling for 10 minutes. Then, the samples were separated by 10% polyacrylamide gel electrophoresis and transferred to polyvinylidene fluoride (PVDF) membranes.

After blocking in 5% skim milk for 1 hour, the membranes were probed with primary antibodies such as DCX (1:500, sc-8066, Santa Cruze), NeuN (1:1000, mab377, Merk), GFAP (1:2000, z0334, Dako), Iba-1 (1:1000, 016-20001, Wako), IL-10R (1:200, sc-365374, Santa Cruze), JAK1 (1:1000, PA5-27583, Invitrogen), *p*-JAK1 (1:1000, 44-422G, Invitrogen), STAT3 (1:2500, MA1-13042, Thermo), *p*-STAT3 (1:1000, MA5-15193, Thermo), BDNF (1:1000, ab108319, Abcam), CREB (1:1000, ab31387, Abcam), *p*-CREB (1:1000, ab32096, Abcam), AQP4 (1:1000, sc-32739, Santa Cruze), p38 (1:1000, 9212S, Cell Signaling), *p*-p38 (1:1000, sc-166182, Santa Cruze), and α-tubulin (1:1000, ab7291, Abcam) antibodies overnight at 4°C. The membranes were washed three times and incubated with an HRP-conjugated anti-goat (1:5000 for DCX), anti-rabbit (1:5000 for GFAP, Iba-1, JAK1, *p*-JAK1, BDNF, *p*-CREB, CREB, and p38), or anti-mouse antibody (1:5000 for IL-10R, STAT3, *p*-STAT3, AQP4, *p*-p38 and α-tubulin) for 45 minutes. The proteins were visualized using an enhanced chemiluminescence (ECL) Advanced Kit. The protein expression level was observed using the FUSION Solo System (Vilber Lourmat, Collegien, France), and band intensity was analyzed with ImageJ version 1.46 (NIH, Bethesda, MD, USA).

### Immunofluorescence staining

Immunofluorescence staining was performed to evaluate the hippocampal neurogenesis-related markers (DCX, BrdU, and NeuN) and newly proliferation astrocytic markers (BrdU and GFAP) in the dentate gyrus and molecules that may be related to astrocyte (GFAP and AQP4) in the hippocampus. The brain tissues were immersed in a 4% PFA solution for 72 hours and subsequently cryoprotected in 10%–30% sucrose solutions for 24 hours each. The brain tissues were embedded in an optical cutting temperature compound with liquid nitrogen and cut into frozen coronal sections (30 μm) using a cryostat (CM3050S, Leica Microsystems, Nussloch, Germany). The frozen brain tissue sections were stored in a cryoprotectant. To block endogenous peroxidase activity, the free-floating sections were immersed in 1% hydrogen peroxide (H_2_O_2_).

The sections were treated with blocking buffer (5% normal chicken serum and 0.3% Triton X-100 in ice-cold PBS) and incubated with a DCX (1:200, sc-8066, Santa Cruze), BrdU (1:200, ab6326, Abcam), GFAP (1:200, Z0334, Dako), AQP4 (1:100, sc-32739, Santa Cruze) or NeuN (1:100, MAB377, Merck) primary antibody overnight at 4°C. After washing with ice-cold PBS, the sections were incubated with a goat anti-mouse IgG H&L (1:400, for AQP4 and NeuN, Alexa Fluor 594-conjugated, ab150116), donkey anti-goat IgG H&L (1:400, for DCX, Alexa Fluor 488-conjugated, ab150129), or goat anti-rabbit IgG H&L (1:400, for GFAP, Alexa Fluor 488-conjugated, ab150077) secondary antibody for 2 hours at room temperature. DCX-, BrdU-, GFAP-, AQP4- or NeuN-stained sections were subsequently exposed to 4ʹ,6-diamidino-2-phenylindole dihydrochloride (DAPI, 1:1000, D9542, Sigma-Aldrich). Immunoreactivity was observed under a fluorescence microscope (× 71, Olympus, Tokyo, Japan). The fluorescence intensity and morphological characteristics of stained astrocytes and microglia (average cell area size per cell and cell number per 0.1 mm^2^) were quantified using ImageJ 1.46 software (NIH, Bethesda, MD, USA). The fold change is computed by dividing the intensity value of the experimental group by the intensity average value of the normal 6-week group, using the recorded intensity.

### Statistical analysis

The data are expressed as the means ± standard deviations. Statistical analysis was performed using GraphPad Prism 9 software (GraphPad, Inc., La Jolla, CA, USA) with normality assessed using the Shapiro–Wilks test. Statistical significance was determined using *t*-tests (unpaired and two-tailed). In all analyses, *p *< .05 was considered significant.

## Results

### SD adversely impacted long-term memory behaviors until early adulthood

**Figure 2.**
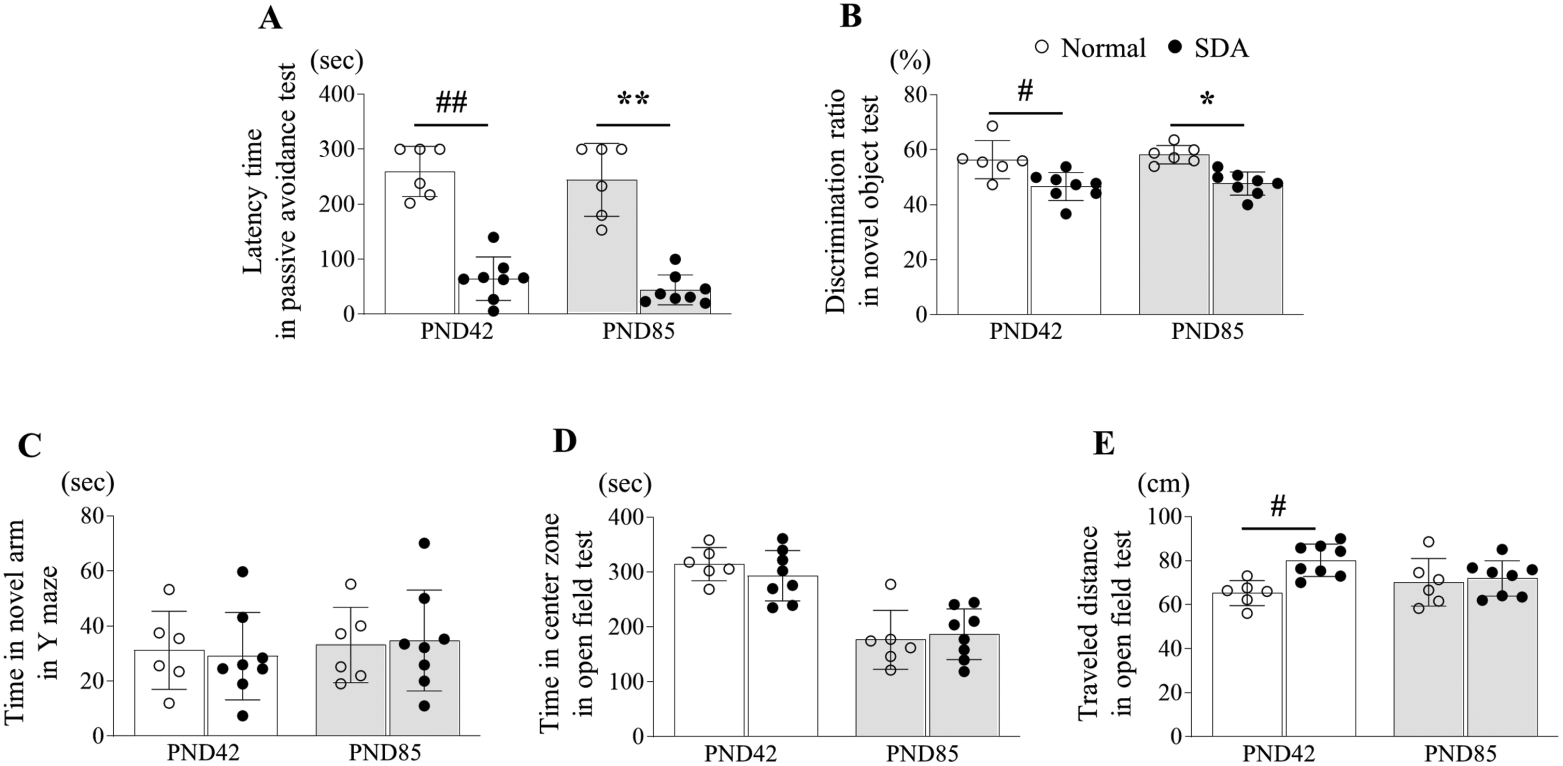
Behavioral changes by sleep deprivation (SD). The behaviors for long-term memory in passive avoidance test (A), cognition in novel object test (B), spatial memory in Y maze test (C), anxiety (D), and locomotor activity (E) in open field test were investigated in mice underwent temporary SD, on days PND42 and PND85, respectively. The data are expressed as the means ± standard deviations (*n* = 6 or 8). #*p* < .05 and ##*p* < .01 compared with the PND42 normal group, *p < .05 and **p < .01 compared with the PND85 normal

Two behavioral tests were conducted to assess long-term memory at two specific time points, PND42 and PND85. The 14-day SD at PND42 significantly reduced the time taken to enter the dark box in the PAT compared to the normal group (*p *< .01). This prolonged latency to enter the dark box persisted at PND85, even after the mice had received adequate opportunity for sleep for 6 weeks (*p *< .01, Figure 2A). Additionally, in the NOT, the 14-day SD significantly decreased the discrimination ratio for a novel object compared to the normal group at both time points of PND42 (*p *< .05) and PND85 (*p *< .05, Figure 2B).

### SD did not impact short-term memory in both adolescence and early adulthood.

Fourteen days of SD in adolescent mice had no effect on short-term memory, as measured by a Y maze test at both PND42 and PND85, when compared to the normal group (Figure 2C). In the OFT, the SD did not change the time spent in a central zone but increased the total distance at PND42 compared to the normal group. These two parameters were not significantly different from the normal group at PND85 (Figure 2, D and E).

### SD diminished hippocampal neurogenesis-related markers until early adulthood.

**Figure 3.**
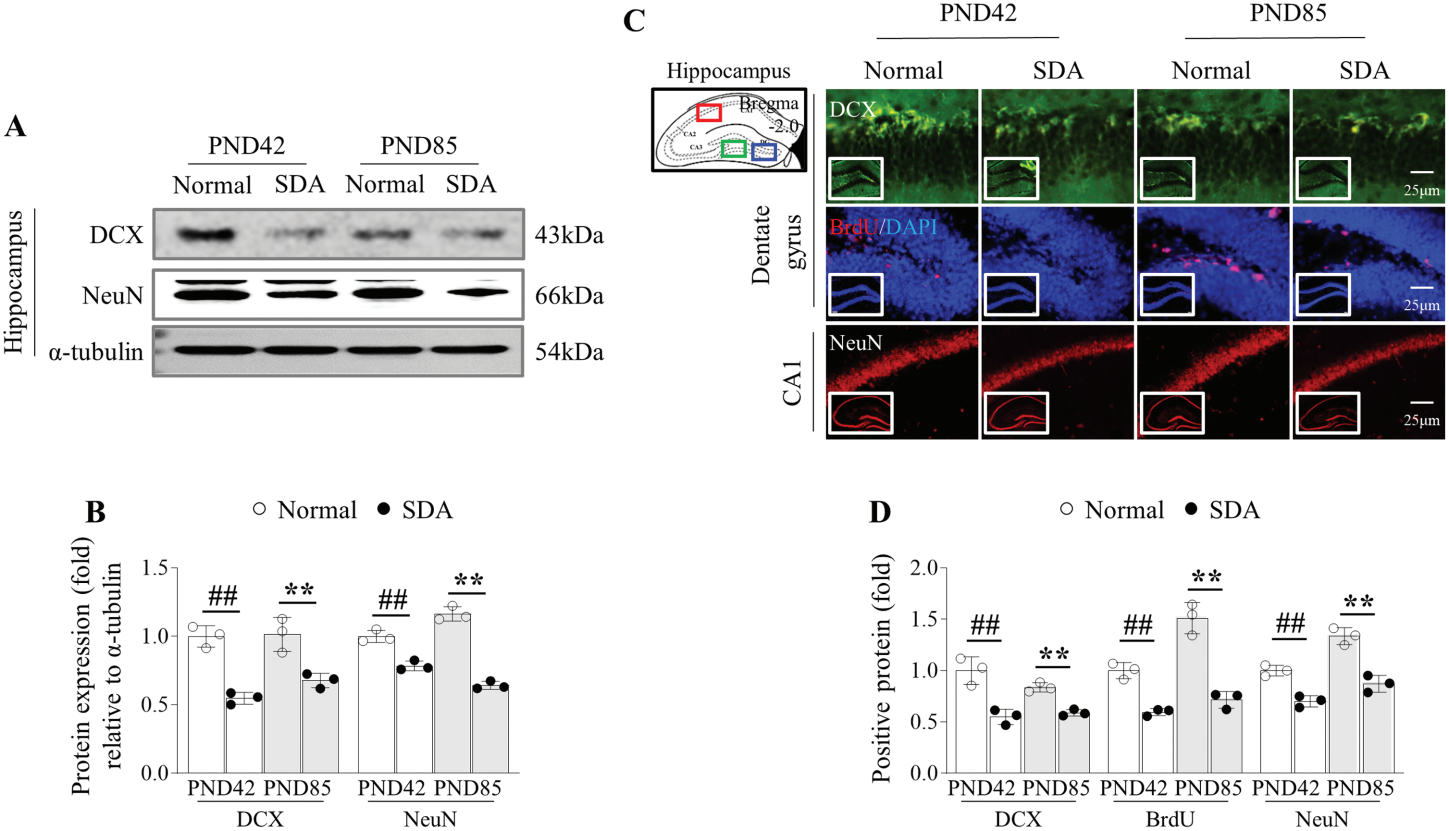
Changes in hippocampal neurogenesis by sleep deprivation. Hippocampal neurogenesis was evaluated by western blot analysis for DCX and NeuN in tissue lysate of the dentate gyrus (A), and its expressions were semi-quantified (B). To verify the neurogenesis using an immunofluorescent staining method, the positive signals to DCX, BrdU, NeuN were analyzed in dentate gyrus (C), and their intensities were semi-quantified (D). The data are expressed as the means ± standard deviations (*n* = 3; evaluated the data in triplicate by pooling the brains of five mice). ##*p* < .01 compared with the PND42 normal group, **p < .01 compared with the PND85 normal group.

The SD in adolescent mice notably altered hippocampal neurogenesis at both PND42 and PND85 as evidenced by the significantly decreased protein expressions of hippocampal DCX and NeuN compared with the normal group (*p *< .01 for both, Figure 3, A and B). Likewise, immunofluorescent staining significantly reduced the intensity of DCX, BrdU, and NeuN positive signals in the hippocampal region at both PND42 (*p* < .01 for all) and PND85 time points (*p *< .01 for all, Figure 3, C and D).

### SD suppressed the expressions of hippocampal astrocytes and microglia until early adulthood

**Figure 4.**
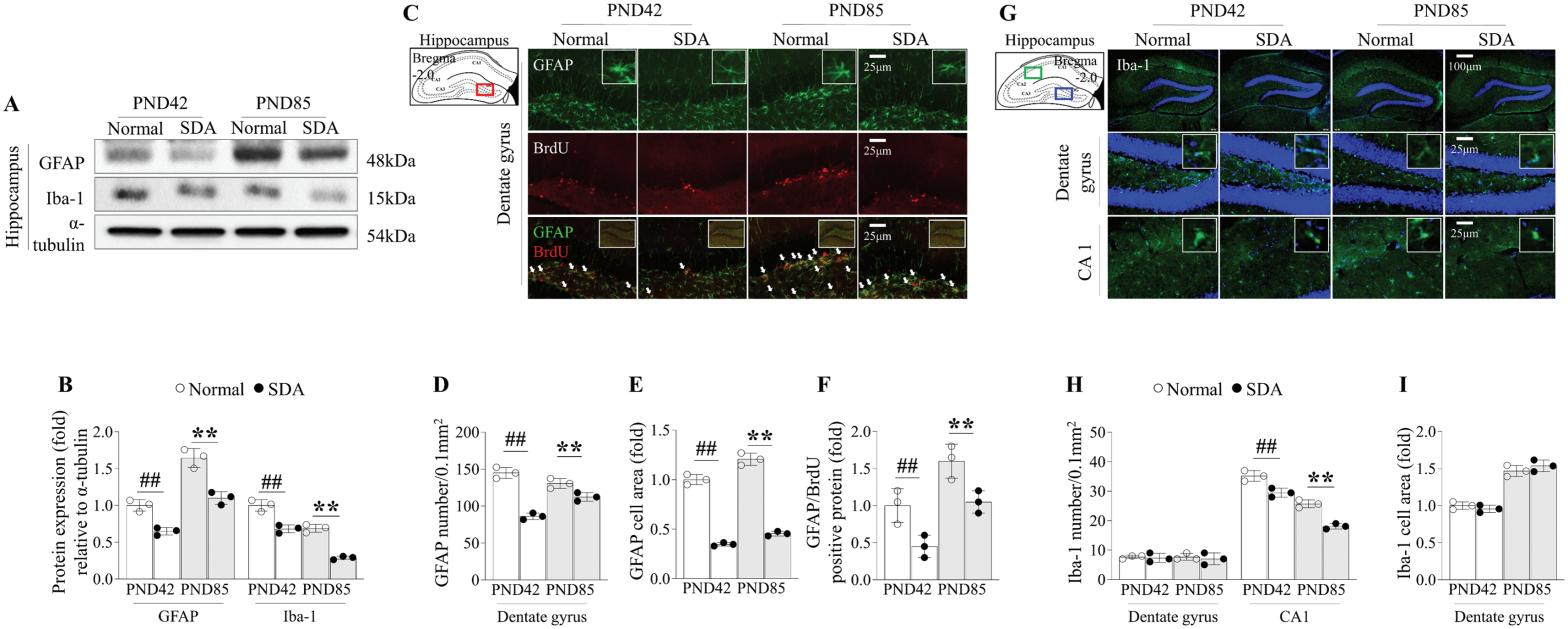
Changes in hippocampal neuroglia expression by sleep deprivation. Activities of neuroglia, including astrocyte (GFAP) and microglia (Iba-1) were evaluated by Western blot analysis in the hippocampus (A), and its expressions were semi-quantified (B). Using an immunofluorescent staining method, the cell number, aera, and morphology of GFAP and double-positive signals of GFAP/BrdU in the dentate gyrus (C) and Iba-1 positive signals in the dentate gyrus and CA1 region (G) were verified. Its intensities were semi-quantified (D to F, H, and I). The data are expressed as the means ± standard deviations (*n* = 3; evaluated the data in triplicate by pooling the brains of five mice). ##*p* < .01 compared with the PND42 normal group, **p < .01 compared with the PND85 normal group

The expression levels of GFAP and Iba-1, classical markers of astrocytes and microglia, respectively, were significantly downregulated in the hippocampus at both PND42 and PND85, in mice from the 14-day SD group (*p *< .01, Figure 4, A and B). Similar to this discovery, astrocyte expressions were greatly decreased at both PND42 and PND85 compared to the normal group. This was seen in the number, intensity, and area of GFAP-positive cells in the dentate gyrus, as well as the BrdU/GFAP double-positive signals. (*p* < .01, Figure 4, C to F). The number, intensity, and area of Iba-1-positive cells in the dentate gyrus show no difference, while microglia in the CA1 region were reduced similarly to protein expression (*p *< .01, Figure 4, G to I).

### SD affected hippocampal molecules that may be related to astrocytes until early adulthood

**Figure 5.**
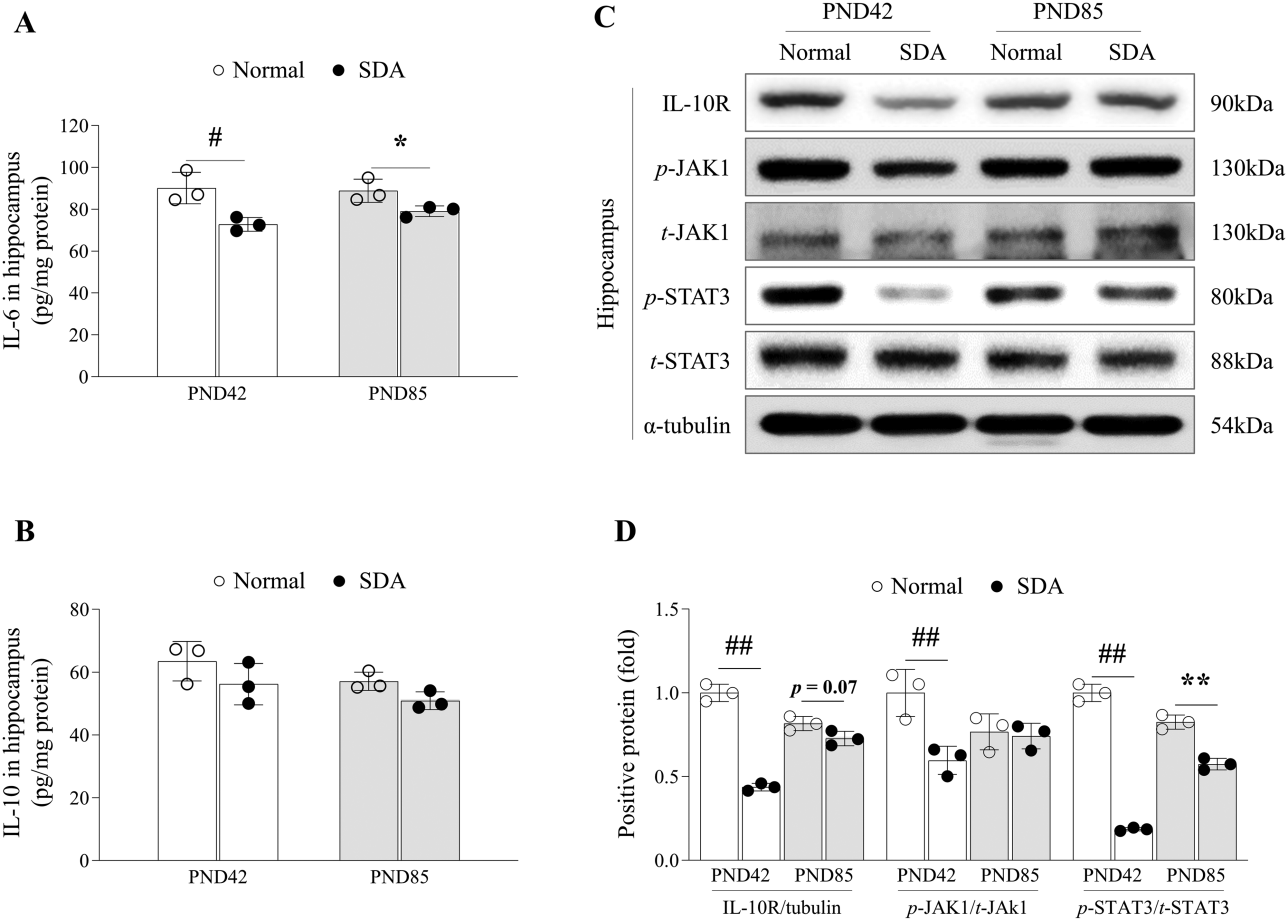
Changes in neuro-immune cytokines and molecules that may be related to astrogenesis in hippocampus by sleep deprivation. Cytokine levels of IL-6 (A) and IL-10 (B) in hippocampal tissue lysate were determined using ELISA. Hippocampal protein expressions of IL-10R, p-JAK1/JAK1, and p- STAT3/STAT3 were measured by Western blotting analysis (C), and its expressions were semi-quantified (D). The data are expressed as the means ± standard deviations (*n* = 3; evaluated the data in triplicate by pooling the brains of five mice). #*p* < .05 and ##*p* < .01 compared with the PND42 normal group, *p < .05 and **p < .01 compared with the PND85 normal group

In comparison to the normal group, mice subjected to a 14-day SD during their adolescence exhibited reduced levels of IL-6 (*p* < .05) and IL-10 (though not statistically significant) in the hippocampus at both PND42 and PND85 time points (Figure 5, A and B), along with decreased IL-10R expression (*p *< .01 and *p* = .07, respectively, Figure 5, C and D). SD did not alter the levels of TNF-α and TGF-β in the hippocampus. Corticosterone levels showed changes only at PND85, differing from the aforementioned results (Figure S1). Additionally, the expression levels of phosphorylated-JAK1 and -STAT3 were significantly suppressed by SD at both PND42 (*p* < .01 for both) and PND85 (*p *< .01 for both, Figure 5, C and D).

### SD inhibited neuronal growth factor and glymphatic molecules until early adulthood

**Figure 6.**
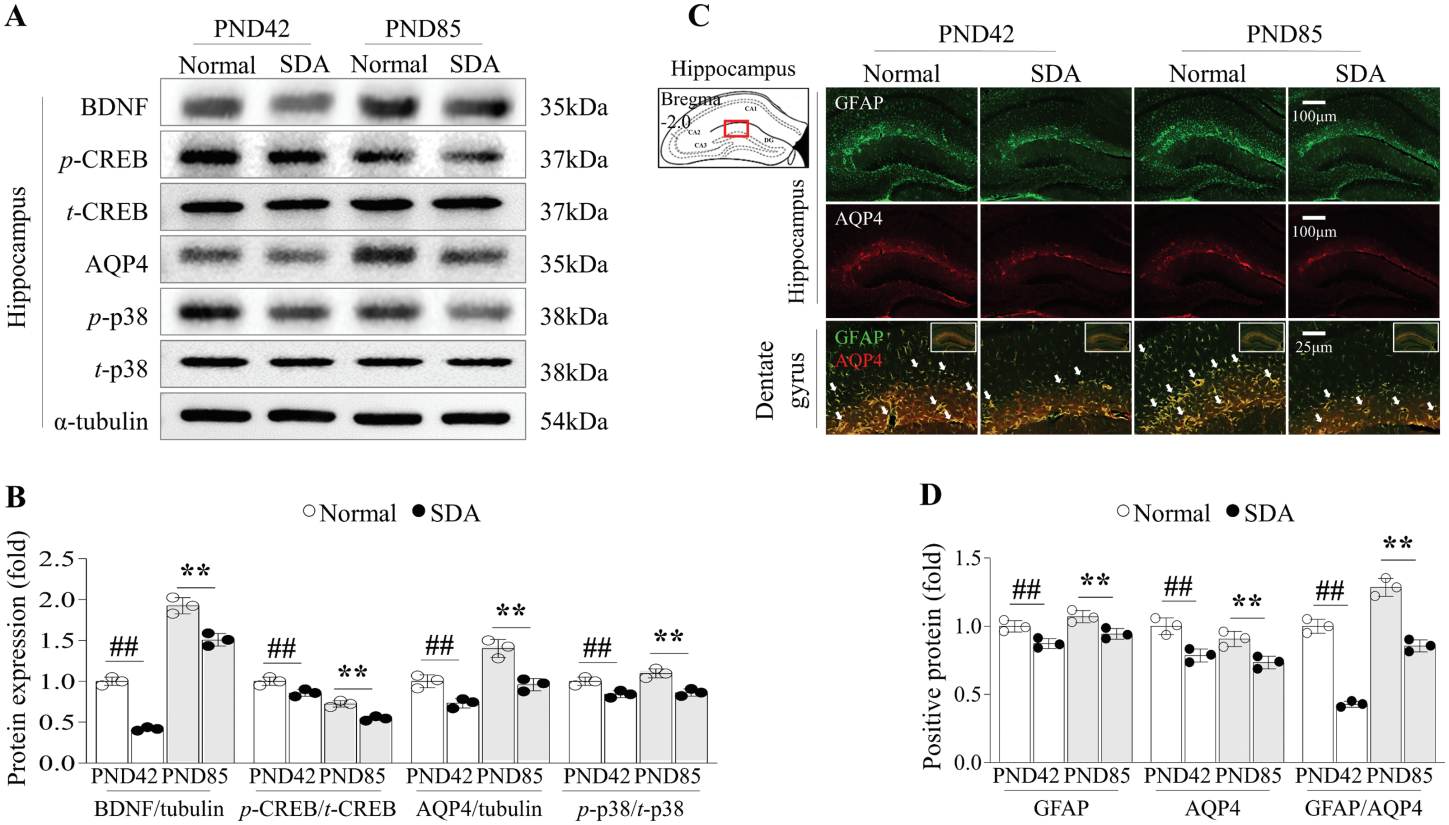
Changes in molecules that may be related to neuronal plasticity and glymphatic activity by sleep deprivation. Hippocampal neuronal activity-related molecules were evaluated by western blot analysis for neuronal plasticity-related molecules (BDNF and p-CREB/CREB) and glymphatic activity-related molecules (Aquaporin 4; AQP4 and p-p38/p38; A) and its expressions were semi-quantified (B). Astrocyte-derived glymphatic activity-related molecules were evaluated by immunofluorescent staining for GFAP and AQP4, and these co-localizations in hippocampus (C), and its positive signals were semi-quantified (D). The data are expressed as the means ± standard deviations (*n* = 3; evaluated the data in triplicate by pooling the brains of five mice). # *p* < .05 and ## *p* < .01 comparing to the PND42 normal group, *p < .05 and **p < .01 comparing to the PND85 normal group.

SD inhibited neuronal growth factor and glymphatic molecules until early adulthood. The 14-day SD in adolescent mice significantly altered two astrocyte-related proteins, BDNF and AQP4, in the hippocampus at both PND42 and PND85 time points (*p* < .01 or *p* < .05 for all), as well as their major transcription factors, phosphorylated CREB and p38, in comparison to the normal group (*p *< .01, western blot, Figure 6, A and B). These findings were consistent with the results obtained from immunofluorescent staining for astrocyte-derived AQP4 (Figure 6, C and D).

## Discussion

It is evident that sleep is inevitable and plays a crucial role in our lives, particularly concerning brain health [30]. The present study investigates how SD alters memory formation. As anticipated, a 14-day period of SD in young mice (PND28) significantly impaired long-term memory (from measurements of PAT and NOT) but not short-term memory (Y maze) at PND42 (Figure 2, A to C). In animal studies, both passive avoidance and NOTs are commonly employed to assess long-term memory, while the Y maze test is utilized to evaluate short-term memory. These tests represent classical experimental tools used to examine memory functions in research contexts [31]. The ages of PND28 and PND42 correspond approximately to 12 to 14 years old and 14 to 16 years old in humans, respectively [32, 33]. During adolescence, memory consolidation and learning processes become exceedingly dependent on sleep [34].

In general, memory can be categorized into two types: short-term and long-term memories. Short-term memory is essential for temporary tasks such as understanding a sentence or following directions [35]. On the other hand, long-term memory stores events, skills, and various types of information acquired over time, making it a critical component of learning abilities as it aids in information retention and retrieval [36]. SD affects both short-term and long-term memory, but its adverse impact is more prominent on long-term memory [37, 38]. A clinical study involving 74 young students (aged 19 to 24 years old) suggested that short-term memory might temporarily improve due to the body’s protective response to 16 hours of SD [39]. Nevertheless, and consistent with our results (Figure 2, A to C), a clinical study reported that sleep-deprived middle school students did not show differences from control students in the letter-number test (assessing short-term memory). However, they exhibited a 20% lower performance in the paired-associate test (evaluating long-term memory) [40]. This asymmetrical phenomenon caused by SD could be associated with a specific brain region. There is substantial evidence that long-term memory is hippocampus-dependent, whereas short-term (working) memory relies primarily on other brain regions such as the prefrontal cortex [41–43].

The crucial role of the hippocampal region in the conversion of short-term memory into long-term memory, known as consolidation, and memory formation has been extensively documented in both animal and human studies [44]. In a clinical study utilizing functional magnetic resonance imaging, enhanced hippocampal memory consolidation was demonstrated through improved recall in word pair tests following post-learning sleep [45]. These processes of long-term memory consolidation and formation are contingent upon cellular processes in the hippocampus, particularly hippocampal neurogenesis [46]. In the present results, 14-day SD in young mice notably altered the intensity signals of DCX, NeuN, and BrdU, the representative markers of hippocampal neurogenesis (Figure 3). Hippocampal neurogenesis arises from the division of stem cells, which are subsequently migrated and stored in cortical regions [47, 48]. Unlike other brain regions, the hippocampus, which is characterized by consistent neurogenesis, has been noted to be particularly susceptible to SD in numerous animal studies [49–51]. These findings strongly corroborate our results, indicating impaired long-term memory, but not short-term memory, due to SD (Figure 2A to C).

Our longitudinal assessments revealed a consistent impairment in long-term memory in young mice (PND42) due to SD that, remarkably, persisted into adulthood (PND85; Figure 2, A and B). In human age equivalence, PND85 corresponds to approximately 20 to 25 years old [32]. These findings suggest that hippocampal dysfunction resulting from sleep insufficiency during youth endures into early adulthood. Additionally, the reduced expression of hippocampal neurogenesis markers (DCX, NeuN, and BrdU) was consistently observed in adulthood as well (Figure 3). These results align with previous research: a 12-day period of SD hindered the generation of new neurons in the dentate gyrus, leading to a reduction in hippocampal volume in a rat model [52, 53]. Furthermore, a clinical study utilizing voxel-based morphometry analysis found that sleep deficiency was linked to a reduction in hippocampal gray matter volume and poorer academic performance among 290 healthy children aged 5 to 18 years [17].

Hippocampal neurogenesis is a multifaceted process originating from a population of neuronal stem cells, involving intricate interactions with glial cells, predominantly astrocytes, and microglia [54, 55]. Astrocytes play crucial roles by providing trophic support, releasing growth factors, and facilitating the integration of new neurons into existing circuits [56, 57]. In contrast, microglia are involved in immune surveillance, modulation of neuroinflammation, and clearance of cellular debris [58]. Previous animal studies have indicated that SD inhibits cell proliferation in the subgranular zone (SGZ) of the hippocampus, primarily consisting of neurons and astrocytes but not microglia [59]. In our study, SD led to significant alterations in protein levels in GFAP and Iba-1, a classical astrocytic and microglial marker, respectively, in the hippocampal region at both PND42 and PND85 (Figure 4, A and B). Iba-1 positive signals in the CA1 region were altered by SD, while the number, intensity, and area of microglia in the dentate gyrus have not changed (Figure 4, G to I). Hippocampal microglial activity is crucial for maintaining the normal structure and function of the astrocytic network during neurogenesis [60, 61]. Patients with astrocytopathy, characterized by the destruction of astrocytes in the brain, often exhibit memory impairment [62, 63]. Indeed, contrary to our findings, several experimental studies have reported the activation of microglia and/or astrocytes under similar conditions [64, 65]. These conflicting pathological phenomena may also be attributed to variations in the quantitative or qualitative aspects of REM SD induced by MMPM [66–69].

During the hippocampal neurogenesis process, astrocytes play a central role by producing neuronal growth factors, such as BDNF, and regulating the brain clearance molecule, such as AQP4 [70, 71]. Inhibition of BDNF from hippocampal astrocytes has been shown to reduce dendrite outgrowth and spine density, leading to memory loss in AD mouse models [72]. In our present study, SD during adolescence significantly reduced the expression of BDNF and its transcription factor (p-CREB) along with co-factors (p-p38) at both PND42 and PND85 (Figure 6, A and B). These sleep deficiency-related changes in astrocytes could be suggested by alterations of phosphorylated JAK1, STAT3 expression, and IL-6 levels in the hippocampus (Figure 5). It is known that cytokines IL-6 and IL-10 and JAK1-STAT3 signaling are involved in hippocampus gene regulation, synaptic plasticity, and astrocyte proliferation [73, 74]. A study found that STAT3 knockdown induced learning impairments and memory deficits, while overexpressing STAT3 rescued cognitive deficits [75]. Immunostaining against BrdU/GFAP double-positive cells further confirmed impaired astrocyte proliferation in the dentate gyrus due to SD at PND85 (Figure 4C). Additionally, AQP4, a major water channel expressed exclusively at the astrocyte endfeet in the brain, was significantly suppressed (Figure 6A to D). Deficiency of hippocampal AQP4 in mice may ultimately lead to long-term memory impairment as a consequence of the loss of long-term potentiation (LTP) [76, 77]. Studies using AQP4 knock-out mice found less LTP in hippocampal slices compared to wild-type mice [78] and demonstrated the crucial role of AQP4 in long-term memory rather than short-term memory [79, 80].

In the present study, we explored the underlying mechanisms related to the negative impact of sleep deficiency in adolescence on long-term memory, extending into adulthood. However, our present study has some limitations. as follows: (1) absence of comparable data examining the impact of sleep deprivation (SD) in adulthood, (2) lack of data comparing other stress inductions to validate the existence or nonexistence of phenomena induced by SD, and (3) necessity for additional long-term follow-up animal studies reflecting mouse-to-human equivalent age, such as until mouse 16 months = approximately human 50 years.

Despite these limitations, to the best of our knowledge, this study represents the first experimental evidence indicating that the impairment of long-term memory in adolescent mice due to sleep deficiency persists into early adulthood. Furthermore, our findings suggest that these impairments are mediated by astrocyte-derived alterations in hippocampal neurogenesis. On the basis of our research, we wish to propose a mechanism by which insufficient sleep during adolescence can result in lasting memory impairment in adulthood. Additionally, we suggest focusing on the regulation of astrocytes as a prospective therapeutic approach.

## Supplementary material

Supplementary material is available at *SLEEP* online.

zsae143_suppl_Supplementary_Figure_S1

## Data Availability

The data underlying this article will be shared on reasonable request to the corresponding author.
