# Supplementary figures and images for: Sleep deprivation in adolescent mice impairs long-term memory till early adulthood via suppression of hippocampal astrocytes
Source: Sleep. 2024 Jun 27;47(10):zsae143. doi: 10.1093/sleep/zsae143 (PMC11467059; doi:10.1093/sleep/zsae143)

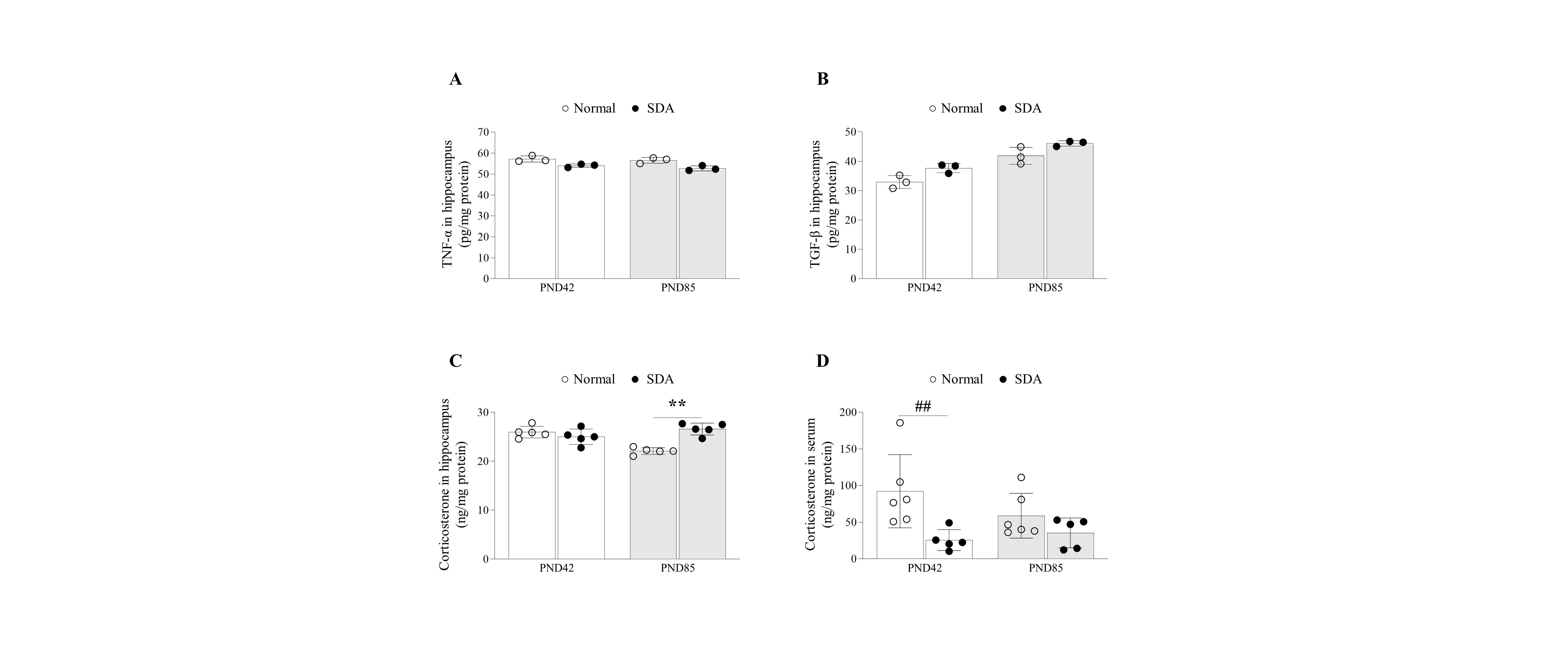

Supplement: zsae143_suppl_Supplementary_Figure_S1 [file zsae143_suppl_supplementary_figure_s1.jpeg]
